# Methylation on RNA: A Potential Mechanism Related to Immune Priming within But Not across Generations

**DOI:** 10.3389/fmicb.2017.00473

**Published:** 2017-03-28

**Authors:** Cynthia Castro-Vargas, César Linares-López, Adolfo López-Torres, Katarzyna Wrobel, Juan C. Torres-Guzmán, Gloria A. G. Hernández, Kazimierz Wrobel, Humberto Lanz-Mendoza, Jorge Contreras-Garduño

**Affiliations:** ^1^Departamento de Biología, Universidad de GuanajuatoGuanajuato, Mexico; ^2^Departamento de Química, Universidad de GuanajuatoGuanajuato, Mexico; ^3^Instituto Nacional de Salud PúblicaCuernavaca, Mexico; ^4^ENES, Unidad Morelia, Universidad Nacional Autónoma de MéxicoMorelia, Mexico

**Keywords:** *Tenebrio molitor*, immune priming, innate immune memory, DNA, RNA, methylation, epigenetics, high-performance liquid chromatography

## Abstract

Invertebrate immune priming is a growing field in immunology. This phenomenon refers to the ability of invertebrates to generate a more vigorous immune response to a second encounter with a specific pathogen and can occur within and across generations. Although the precise mechanism has not been elucidated, it has been suggested that methylation of DNA is a cornerstone for this phenomenon. Here, using a novel method of analytical chemistry (a reversed-phase liquid chromatography procedure) and the beetle *Tenebrio molitor* as a model system, we did not find evidence to support this hypothesis taking into account the percentage of methylated cytosine entities in DNA (5mdC) within or across generations. However, we found a lower percentage of methylated cytosine entities in RNA (5mC) within but not across generations in immune priming experiments with adults against the bacteria *Micrococcus lysodeikticus* and larvae against the fungus *Metarhizium anisopliae*. To our knowledge, this is the first report suggesting a role of differential methylation on RNA during immune priming within generations.

## Introduction

Recent evidence suggests that invertebrates have properties that resemble vertebrate immune memory (Contreras-Garduño et al., 2016; Milutinović and Kurtz, 2016). The process has been termed immune priming, and it refers to enhanced protection (i.e., resistance, immune response, and survival) to a pathogen or parasite in a second encounter after a first specific exposure (Kurtz and Franz, 2003; Little and Kraaijeveld, 2004; Kurtz, 2005). This phenomenon has been reported in ctenophores, sponges, mollusks, crustaceans and insects, among others (Milutinović and Kurtz, 2016). Within generations, immune priming is strain or species specific (Roth et al., 2009) and long lasting (it can persist across different life developmental stages; Thomas and Rudolf, 2010). In addition, immune priming not only occurs within generations but also across generations: parents can protect their offspring against the same parasites or pathogens that they confronted (Sadd and Schmid-Hempel, 2007). In bumble bees (*Bombus terrestris*), when the parental colony was either injected with lipopolysaccharides diluted in Ringer’s solution (primed group) or injected with Ringer’s solution (Control group); the male offspring derived from the primed group exhibited more phenoloxidase activity than the control group offspring (Moret and Schmid-Hempel, 2001). In another study, female bumblebees that were challenged with heat-killed bacteria produced eggs with more antimicrobial activity than eggs of non-challenged control females (Sadd and Schmid-Hempel, 2007). Immune priming across generations has been documented in both wild and laboratory populations (Tate and Graham, 2015) and such changes can be transmitted by either insect mothers or fathers (Roth et al., 2009). This suggests that some information is passed from parents to offspring but the mechanism remains understood.

As mentioned above, the molecular basis underlying immune priming within and across generations remains poorly understood (Brehélin and Roch, 2008; Rodrigues et al., 2010; Pope et al., 2011; Mikonranta et al., 2014; Contreras-Garduño et al., 2015; Tate and Graham, 2015); but DNA methylation has been proposed as cornerstone (Jokela, 2010; Ottaviani, 2015). However, experimental evidence of differential epigenetic mechanisms of immune priming is lacking, both within and across generations (Eggert et al., 2014; Norouzitallab et al., 2015; Ottaviani, 2015). Epigenetic mechanisms refer to stimuli-triggered events that generate variation in gene expression without changing the DNA sequence by favoring, reducing or inhibiting gene expression within or across generations (Suzuki and Bird, 2008). One of the most important epigenetic mechanisms involves DNA methylation, which occurs at carbon 5 of the pyrimidine ring in cytosine residues (5mdC) (Suzuki and Bird, 2008; López-Torres et al., 2011). DNA methylation is highly variable in eukaryotes and in some insect species, such as the beetle *Tribolium castaneum*, in which no detectable nuclear DNA methylation has been found (Zemach et al., 2010) whereas in another beetle, *Nicrophorus vespilloides*, there is strong evidence (Cunningham et al., 2015). Another recently discovered mechanism is a covalent modification that takes place in different types of RNA (5mC); but the biological significance of this mechanism remains unknown (Mattick et al., 2009; Motorin et al., 2010; Yanez-Barrientos et al., 2013; Yan et al., 2015; Chen et al., 2016). Despite the traditional view that the function of RNAs is to act as messengers between DNA and protein synthesis, recent evidence suggests that RNAs are also involved in the regulation of genome organization and gene expression (Morris and Mattick, 2014; Chen et al., 2016). Therefore, it is important to assess methylation in both DNA and RNA during the immune priming phenomenon.

We used the beetle *Tenebrio molitor* as a model system to assess global DNA and RNA methylation after a second challenge during immune priming. This species exhibit immune priming across generations (Moret, 2006; Zanchi et al., 2012). We used a novel procedure in analytical chemistry based on hydrolysis of DNA/RNA to nucleosides followed by selective fluorescent labeling of cytosine moieties with 2-bromoacetophenone and reversed-phase liquid chromatography separation (López-Torres et al., 2011; Yanez-Barrientos et al., 2013). This procedure allows for high precision and accuracy in the analysis of sub-microgram amounts of nucleic acids and it is validated by other methods to measure methylated DNA or RNA (López-Torres et al., 2011; Yanez-Barrientos et al., 2013). We found that the priming group had higher survival upon re-challenge than the control group, both within and across generations. However, we only found significant differences in RNA methylation within the generation exposed to the pathogen, and no evidence of methylated DNA was found. Furthermore, we investigated whether DNA and/or RNA methylations were found only in adult insects or were also present in larvae, and if methylation was related to the type of pathogen (bacteria or fungus) used as challenge. Larvae of *T. molitor* in the immune priming group exposed to an entomopathogenic fungi showed lower levels of RNA methylated 24 h after the second challenge compared to control group, but no evidence of DNA methylation was found in any group. To our knowledge, this is the first documented link between RNA methylation and immune priming in invertebrates.

## Materials and Methods

### Beetle Source and Rearing Conditions

The mealworm colony was obtained from several sources, including Mexican pet stores and research cultures from different institutions: Colegio de Postgraduados and from the Centro de Investigación y Estudios Avanzados del Instituto Politécnico Nacional. Obtained larvae (about 15,000) were reared until adulthood and resultant adults were bred together for three generations (over an 18 months period) before use in our experiments. The colony was maintained at 27 ± 0.5°C and a 12:12 h L:D inverted photoperiod (Márquez-García et al., 2016). Food *ad lib* consisted of bran and corn meal (1:1) with fresh apple slices added every other day (Márquez-García et al., 2016). The food was sterilized (125 ± 2°C for 15 min) to avoid infections, which could bias the immune priming protocols. Experimental animals were also fed sterilized food during all tests. Pupae from the colony were collected and sexed daily according to Bhattacharya et al. (1970).

At least two replicates per experiment were carried out, which consisted in using fresh prepared immune challenge (fungus or bacteria) and insects of similar size, but that belonged to different cohorts reared in different containers. Insects were used only once per replicate. An immune challenge is referred as the injection of an immune elicitor (i.e., one injection of fungus conidia or bacterial cells) or the injection of the solvent in which the immune treatment was diluted. Immune priming was tested by comparing survival between homologous challenges (two similar immune challenges) against controls (PBS and Control groups, see below).

### Experiment 1: Within Generation Immune Priming in Adults

**FIGURE 1:**
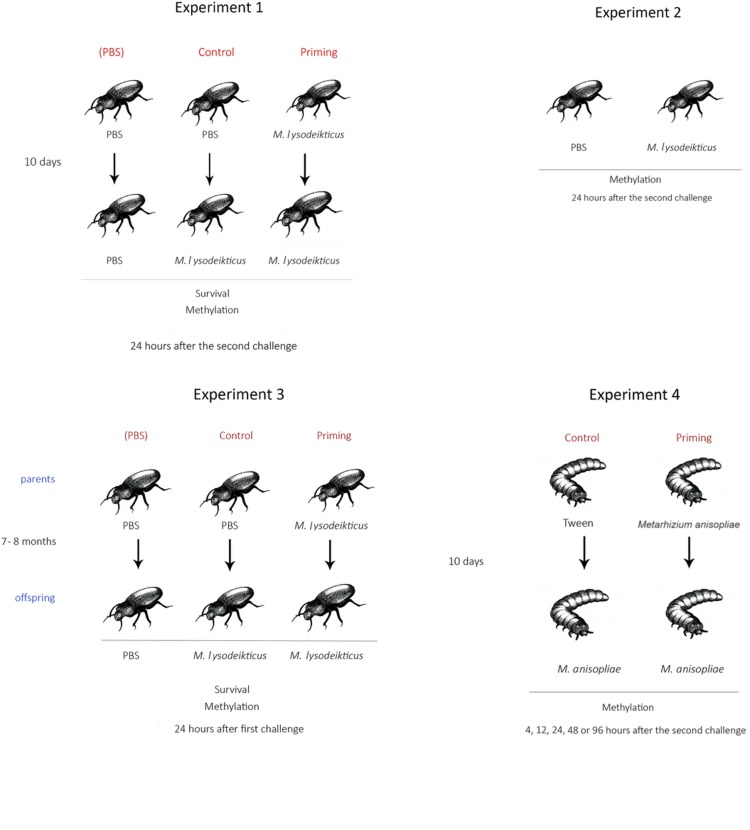
**Schematic representation of the experimental design.** Image courtesy of Ángela Arita.

Adult beetles of each sex were injected through the intersegmental membrane of the coxa of the third pair of legs with a sterilized micro syringe (10 μL, Hamilton). Three treatments were used (**Figure 1**; Experiment 1): (1) the PBS group was injected twice with 1 μL of Phosphate Buffer Saline (Sigma) and served as control beetles with no previous pathogen contact; (2) the Control group received 1 μL of PBS and then 1 μL of *Micrococcus lysodeikticus* (Sigma) (1 mg/mL) diluted in PBS, and served as the standard immune response to the pathogen; and (3) the Priming group was injected with 1 μL of 0.25 mg/mL of *M. lysodeikticus* and then received 1 μL of *M. lysodeikticus* (1 mg/mL); to determine the effect on the immune system with a previous exposure to the pathogen. For all treatments, the first injection was performed 3 days after the beetles emerged as adults while the second injection was performed 10 days later, when the beetles were 13 days old. To assess the effects of the treatments, the percentage of surviving insects was calculated 24 h after the second challenge.

### Experiment 2: Effect of PBS or Immune Challenge on DNA and RNA Methylation

To ensure that differential methylation observed between treatments in the experiment described above was due to immune priming (memory) and not to the immune challenge (only activation of immune response), we carried out another experiment in which adults were injected once with either 2 μL of *M. lysodeikticus* (1 mg/mL) (*n* = 25) or only with 2 μL of PBS (*n* = 25) (**Figure 1**; Experiment 2).

### Experiment 3: Across-Generation Immune Priming in Adults

To assess the possibility of across-generation immune priming in adults, 3-day old adults received an injection of either PBS or *M. lysodeikticus* (0.25 mg/mL). When beetles were 13 days old, they were paired within treatments (PBS-male with PBS-female or *M. lysodeikticus*-male with *M. lysodeikticus*-female) and then held in Petri dishes (100 mm diameter, Corning) to mate and oviposit. Eggs were obtained 5 days later, and resulting offspring were reared until adulthood (7–8 months later). The same diet as their parents was provided when reared. Offspring were then subjected to an immune challenge. Offspring from PBS parents received either an injection of PBS or *M. lysodeikticus*. Offspring of parents that had been injected with a priming dose of 0.25 mg/mL *M. lysodeikticus* received an injection of 1 mg/mL of *M. lysodeikticus* (**Figure 1**; Experiment 3).

### Experiment 4: Within Generations Immune Priming in Mealworm Larvae after Exposure to Fungus

**FIGURE 2:**
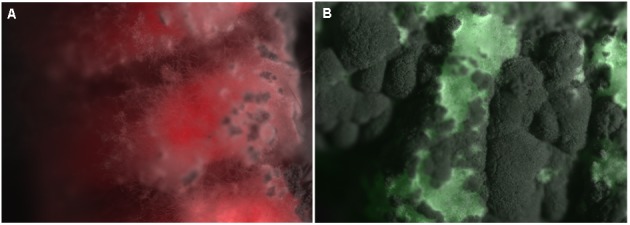
**Larvae of *T. molitor* infected with *Metarhizium anisopliae* strains Ma10mCherry (A)** or Ma10GFP **(B)**. Figure shows the larvae cuticle covered by the fungus. Both strains contain the same genotype except for the overexpression of mCherry or Green Fluorescent Protein (GFP) under the GPDa promoter, respectively. Images were acquired using a Nikon Optiphot-2 microscope with a SPOT RT- Color camera and SPOT 4–6 software (Diagnostic instruments). GFP and mCherry fluorescence was detected using the recommended filters (Chroma Technology). Larvae of dual challenges did not show evidence of hyphae growing on its body 9 days after the first challenge, just before inoculation with 200 conidia of Ma10GFP.

Since we had previously demonstrated within generation immune priming in *T. molitor* larvae following exposure to the fungus *M. anisopliae* (Medina et al., unpublished), here we examined the degree of RNA/DNA methylation in larvae at different time points (4, 12, 24, 48, and 96 h) after the second challenges with the fungus. Two treatments were used: (1) a Control group, which represents the response to the full fungal dose, was injected with 1 μL of Tween 80 (0.01%) and again 10 days later with 1 μL of *M. anisopliae* conidia diluted in Tween 80 to 200 conidia/μL, and (2) a Priming group, which was injected with 1 μL of *M. anisopliae* diluted in Tween 80 to 5 conidia/μL and then 10 days later was injected with 1 μL of *M. anisopliae* diluted in Tween 80 to 200 conidia/μL (**Figure 1**; Experiment 4). For the priming treatment, the first challenge was carried out with the fungal strain Ma10mCherry (**Figure 2**) and the second challenge with Ma10GFP (**Figure 2**). A pilot experiment did not find differences in survival in larvae challenged with 5 conidia/μL between Ma10mCherry vs. Ma10GFP (Gehan = 3.6; *p* > 0.05) or challenged with 200 conidia/μL between Ma10mCherry vs. Ma10GFP (Gehan = 2.0; *p* > 0.05). The use of these two strains, which did not differ in mortality rates, then let us to confirm that fungus were cleaned before the second challenge. This is that larvae (*n* = 30) inoculated with 5 conidia of Ma10mCherry showed no sign of infection 9 days later, just before inoculation with 200 conidia of Ma10GFP. This suggests that the survival in dual challenges with the fungus was not due to an overlap of infection (immune enhancement), but possibly to immune memory.

Larvae were injected through the pleural intersegmental membrane between the sixth and seventh abdominal segments using sterilized micro syringes (10 μL Hamilton syringe). After the second challenge, larvae were placed individually into 12-well plates cells (Corning) with sterilized food. Mortality was assessed daily for 10 days and we corroborated the growth of mycelia in mealworm cadavers (**Figure 2**).

### Measurement of DNA and RNA Methylation via Liquid Chromatography

For Experiments 1 and 3, insects were frozen at -70°C 24 h after the second challenge to assess methylation, based on the expectation that the immune reaction would be fully expressed within 24 h of the immune challenge (Haine et al., 2008; Johnston et al., 2014) and the PBS group was not included in this comparison. In Experiment 4 larvae were frozen at 4, 12, 24, 48, and 96 h after the second challenge to follow the time course of methylation. In Experiment 2 adults were frozen 24 h after activation of immune response.

Nucleic acids were extracted from each individual according to the method of Aljanabi and Martinez (1997). The extracts were analyzed by reversed-phase liquid chromatography based on fluorescent labeling of cytosine and methyl-cytosine moieties with 2-bromoacetophenone (HPLC-FLD; López-Torres et al., 2011; Yanez-Barrientos et al., 2013). The analytical procedure applied here enables the determination of both parameters in a single sample of nucleic acids extract from an individual insect (not less than 1 μg of DNA + RNA) and in one chromatographic run (Yanez-Barrientos et al., 2013).

Specifically, we dissected each insect and used only fat body and thorax muscles. After removal, tissues were homogenized by repeated pipetting with 400 μL of buffer containing sodium chloride 0.4 M, EDTA 2 mM, and Tris-HCl 10 mM at pH 8 according to Aljanabi and Martinez (1997). After homogenization, 40 μL of sodium dodecyl sulfate 20% (m/v) and 8 μL of proteinase K (20 mg/mL) were added. This mixture was incubated for 1 h at 60°C, then 300μL of sodium chloride 6 M was added and the material was centrifuged at 10,000 ×*g* for 30 min. The supernatant was collected, 500 μL of isopropanol were added and the mixture held at -20°C for 1 h to complete nucleic acid precipitation. Samples with precipitated nucleic acids were centrifuged (10,000 ×*g*, 20 min, 4°C) and the supernatant was discarded. The pellet from centrifugation was washed with 500 μL of ethanol 70% and dried.

To estimate the purity of extracts and the amount of nucleic acid (DNA + RNA), 10 samples from individual control insects were pooled and processed as described above, reconstituting the final pellet in 500 μL of deionized water. Samples were analyzed via absorption spectrum, in the wavelength range of 220–350 nm (Spectronic 3000, Milton Roy, Co. Ltd). Absorbance ratio (of 260/280 nm) obtained on average was 1.8, which indicates acceptable purity for nucleic acids (Aljanabi and Martinez, 1997). The amount of total nucleic acid (DNA + RNA) was determined by measuring absorbance at 260 nm; accordingly, the evaluated mass of nucleic acids extracted from single insect was not less than 1 μg.

For hydrolysis of nucleic acids to nucleosides, the pellet from each individual insect was brought to a volume of 42 μL with deionized water and then 5 μL of the hydrolysis buffer (acetic acid 200 mM, glycine 200 mM, magnesium chloride 50 mM, zinc chloride 5 mM, calcium chloride 2 mM, pH 5.3) was added. The sample was then incubated with 0.2 μL of DNAse I (10 U/μL) and 0.2 μL of nuclease P (1.1 U/μL) at 37°C overnight. Reactions were stopped by heating the sample in boiling water for 5 min and then cooling it rapidly on ice. Afterward, sodium hydroxide (5 μL, 100 mM) was added, together with 0.2 μL of alkaline phosphatase (1 U/μL), and the sample was again incubated at 37°C for 2 h. Samples containing DNA and RNA nucleosides were stored at -20°C until needed.

The analytical procedure used in the present study (López-Torres et al., 2011; Yanez-Barrientos et al., 2013) requires derivatization of cytosine moieties with 2-bromoacetophenone. For this purpose, an aliquot of hydrolyzed sample was placed in a 350 μL insert in the auto sampler amber vial, the volume was brought to 60 μL with deionized water, and the sample was evaporated (SpeedVac Vacufuge plus, Eppendorf, 2 000 rpm, 60°C, 20 min). The residue was reconstituted in 130μL DMF anhydrous with the addition of 5μL glacial acetic acid. Afterward, 20 μL of 2-bromoacetophenone (0.5 M in DMF anhydrous), 2-bromoacetophenone sodium sulfate anhydrous (0.5 M in DMF) sodium sulfate anhydrous was added and the mixture was heated at 80°C for 90 min; at this stage, the samples were protected from light. The vials were placed in the auto sampler compartment, diluted 1:1 with deionized water and injected to the column (4 μL). Column temperature was kept at 30°C and gradient elution with four mobile phases [water (A), acetonitrile (B), TFA 0.4% m/v (C), methanol (D)] was as follows: 0–2 min 62% A, 5% B, 13% C, 20% D; 2–9 min 49% A, 10% B, 13% C, 28% D; 9–13 min 47% A, 12% B, 13% C, 28% D; 13–16 min 12% A, 15% B, 13% C, 60% D with the total flow rate 0.35 mL min^-1^. The derivatization reaction was selective for C, 5mC, dC, and 5mdC. Using fluorimetric detection with excitation/emission wavelengths at 306/378 nm, respectively, these four compounds could be determined with excellent selectivity and sensitivity.

We defined global methylation status as the percentage of methylated cytosine moieties with respect to all cytosine residues present in DNA or RNA; therefore, it was not necessary to control the amount of nucleic acid in the sample analyzed. However, samples should not be under 100 ng (Yanez-Barrientos et al., 2013). As described previously (López-Torres et al., 2011; Yanez-Barrientos et al., 2013), the percentage of methylation can be assessed using external calibration. Calibration solutions for DNA contained different molar ratios of 2′-deoxycytidine to 5-methyl-2′deoxycytidine [MR(dC/5mdC)], covering percentages of cytosine methylation from 0.12 to 9.5%. For RNA, the calibration samples contained different molar ratios of cytidine to 5-methylcytidine [MR(C/5mC)] covering percentages of cytosine methylation from 0.24 to 2.37%. These solutions were injected into the chromatographic system and peak areas for cytidine [A(C)], methylcytidine [A(5mC)], 2′-deoxycytidine [A(dC)], and 5-methyl-2′deoxycytidine [A(5mdC)] were measured. Linear regression functions were obtained as follows: for RNA: A(C)/A(5mC) = 1.070 MR(C/5mC) + 2.261 (*R*^2^ = 0.9999) and for DNA: A(dC)/A(5mdC) = 1.073 MR(dC/5mdC) + 0.182 (*R*^2^ = 0.9999). For individual insect extracts, ratios between areas of chromatographic peaks A(dC)/A(5mdC) and A(C)/A(5mC) were measured, respective molar ratios were obtained from calibration functions and the percentage methylation of nucleic acids were calculated from the following equations: % DNA = [1/(1 + MR(dC/5mdC)]∙100%; % RNA = [1/(1 + MR(C/5mC)]∙100%.

**FIGURE 3:**
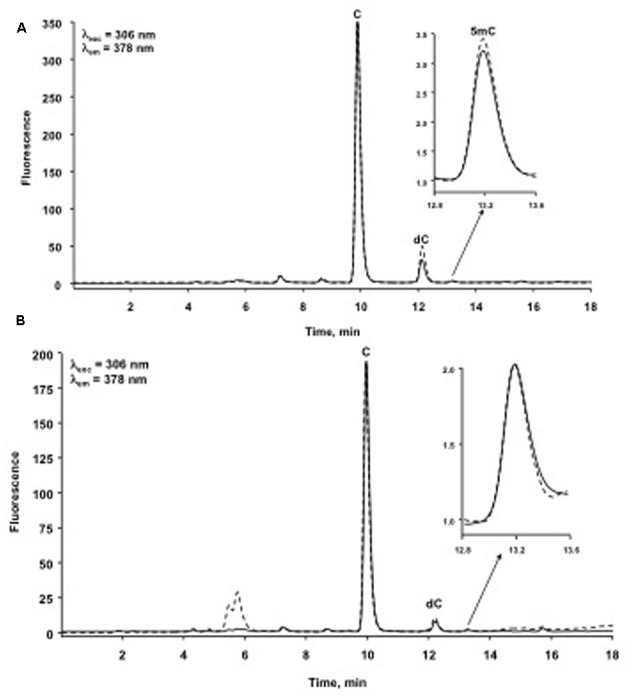
**Typical chromatograms of immune priming within generations obtained from one Control insect (dashed line) and one Priming insect (solid line).** The full-scale mode represents **(A)** one insect per treatment from the experiment of immune priming within generations; **(B)** one insect per treatment from the experiment of immune priming across generations. C, cytidine; dC, 2′-deoxycytidine; 5mC, 5-methylcytidine.

For clarity of presentation, chromatograms in **Figure 3** were processed from their original form by normalization (software option “full-scale mode”); by doing so, cytidine peak has the same area in each chromatogram and any change in 5-methylcytidine can be clearly observed among different samples.

### Statistical Analyses

We compared the likelihood of survival 24 h after a second challenge between Priming, PBS and Control groups by using a Chi-square test. To compare rates (%) of methylation at 24 h after the second challenge, we used a Student *t*-test. In all cases, we confirmed the assumption of normality and variance homogeneity. All analyses were carried out using STATISTICA (StatSoft). Sample sizes are provided in figure legends.

## Results

### Experiment 1: Within Generation Immune Priming in Adults against Bacteria

**FIGURE 4:**
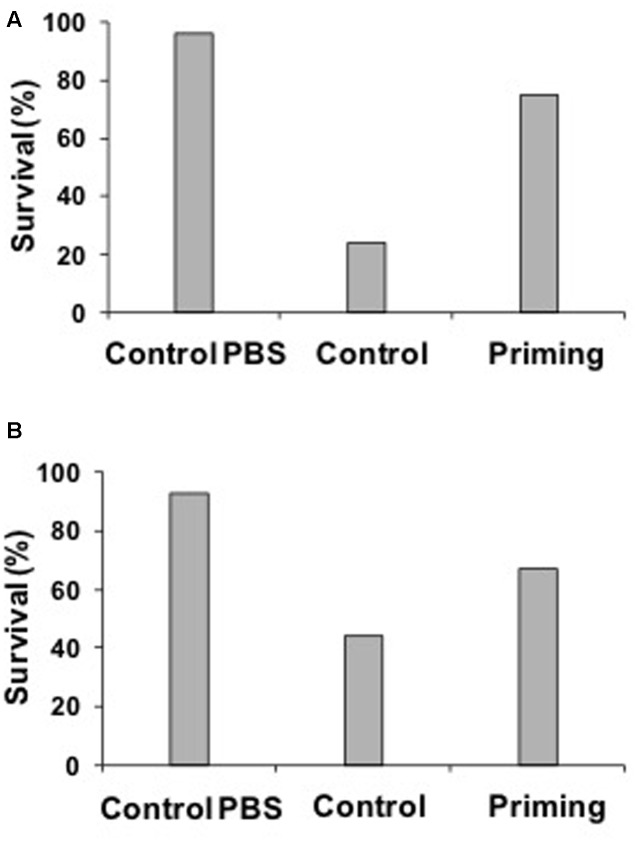
**Percentage of females (A)** or males **(B)** that survived according to treatment 24 h after the second challenge. Sample sizes are shown in the text. Taking into account the females **(A)**, after 24 h of the second challenge it was more likely that PBS (live females: 96%, dead females: 4%, *n* = 92) and Priming group survived (live females: 75%, dead females: 25%, *n* = 95) than the Control group (live females: 24%, dead females: 76%, *n* = 92; *X*^2^ = 68.34, *p* < 0.0001). A similar result was found in males **(B)**, after 24 h of the second challenge it was more likely that PBS (live males: 93%, dead males: 7%, *n* = 95) and Priming group (live males: 67%, dead males: 33%, *n* = 51) survived than males of the Control group (live males: 44%, dead males: 56%, *n* = 112; *X*^2^ = 43.48, *p* < 0.0001).

Our results revealed that both, males and females showed immune priming (**Figures 4A,B**), and therefore data were pooled. Less percentage of adults survived in the Control group (surviving insects: 35%, dead insects: 65%, *n* = 200) compared with the PBS group (surviving insects: 94%, dead insects: 6%, *n* = 200) and Priming group (surviving insects: 72%, dead insects: 28%, *n* = 200; *X*^2^ = 52.90, *P* < 0.0001).

**FIGURE 5:**
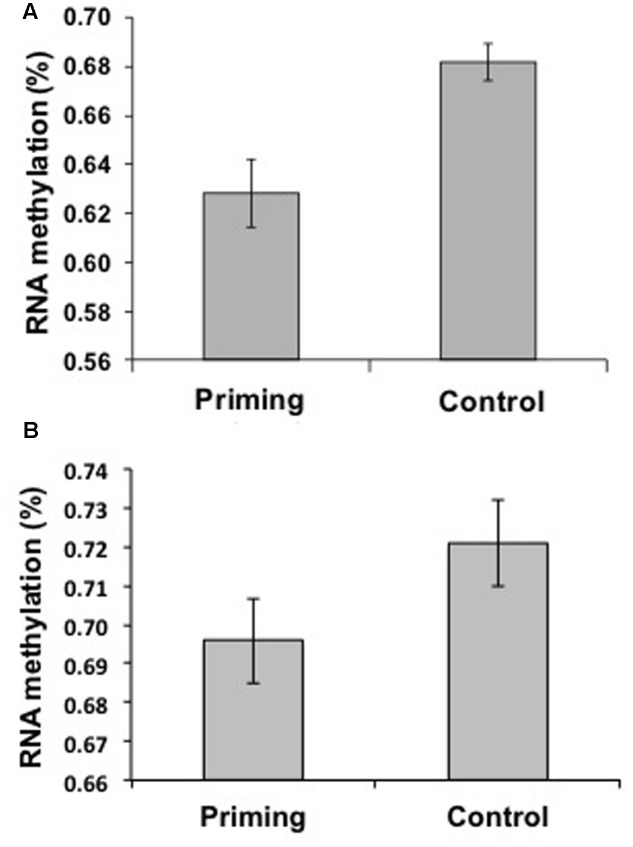
**Levels of RNA Methylation by treatment within generations in *Tenebrio molitor*. (A)** Adult primed insects (*n* = 25) had a lower percentage of methylation than did Control animals (*n* = 20) when challenged with *M. lysodeikticus*, as measured 24 h after the second challenge. **(B)** Primed larvae (*n* = 19) had a lower percentage of methylation than did Control animals (*n* = 18) when challenged with *M. anisopliae*, as measured 24 h after the second challenge. The means ± standard errors of the percentages of methylation among treatments are shown.

Neither the control nor the priming parents exhibited 5mdC (indicative of DNA methylation), but we found evidence of 5mC (indicative of RNA methylation). The percentage of total RNA methylation was lower in the Priming group (0.62 ± 0.014%, *n* = 25) compared to the Control group (0.68 ± 0.007%, *n* = 20; *t* = 3.11, *p* = 0.003; **Figure 5A**).

### Experiment 2: Effect of PBS or Immune Challenge on DNA and RNA Methylation

As a potential confounding variable of the above experiment is that differential methylation could be due to immune activation rather than immune memory, we compared methylation on DNA and RNA 24 h after an immune challenge. Results revealed no significant differences in RNA methylation between the immune-challenged (0.65 ± 0.01%, *n* = 25) and control for challenge (0.66 ± 0.008%, *n* = 25; *t* = -0.35, *P* = 0.7) 24 h after immune activation.

### Experiment 3: Across Generation Immune Priming in Adults against Bacteria

When parent beetles were injected with PBS and the offspring injected with PBS (no pathogen exposure), offspring had the highest survival 24 h after injection (live: 96%, dead: 4%) compared to offspring in the Priming group, whose parents were injected with *M. lysodeikticus* (live: 52%, dead: 48%) or than the Control group, whose parents were not injected but their offspring injected (live: 5%, dead: 95; *X*^2^ = 82.90, *P* < 0.0001). However, the Priming group survived better than the Control group (*X*^2^ = 40.58, *P* < 0.0001).

The DNA methylation marker (5mdC) was absent in the beetle progeny in the across generation experiment, but we found the RNA methylation marker (5mC). However, compared to effect of immune priming within a generation, the degree of RNA methylation (%) found in the across generation experiment did not differ significantly between the Priming group (0.62 ± 0.018%, *n* = 25) and the Control group (0.64 ± 0.031%, *n* = 20; *t* = 0.66, *P* = 0.4).

### Experiment 4: Within Generation Immune Priming in Mealworm Larvae after Exposure to the Fungus *M. anisopliae*

As in Experiment 1, we found differential methylation on RNA in adults against *M. lysodeikticus*, this result may be due to the specific beetle life stage against bacteria but not a more general phenomenon of immune priming. Hence, we used larvae injected with the fungus *M. anisopliae* to assess if differential methylations on RNA were observed independently of the insect life-stage and pathogen used. The percentage of 5mC (indicative of RNA methylation) was lower in the Priming group (0.69 ± 0.01%, *n* = 19) than in the Control group (0.72 ± 0.008%, *n* = 18; *t* = 2.74, *P* = 0.008; **Figure 5B**). In addition, as showed previously in adults against *M. lysodeikticus*, we did not find methylation on 5mdC (indicative of DNA methylation).

**FIGURE 6:**
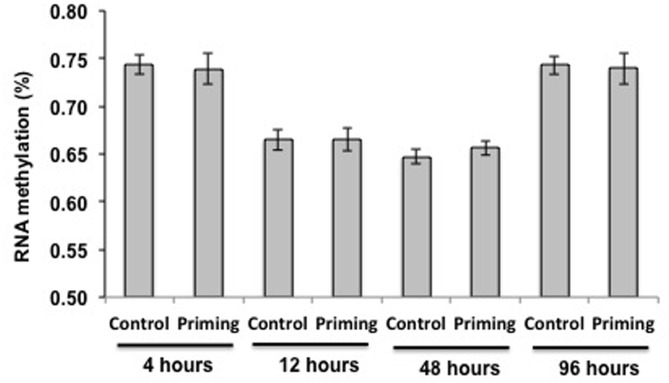
**RNA methylation for treatments and time periods in larvae against *M. anisopliae*.** Tissue was extracted at 4 (Control *n* = 20 and Priming = 20), 12 (Control *n* = 23 and Priming = 19), 48 (Control *n* = 19 and Priming = 20) and 96 h (Control *n* = 20 and Priming = 20). The means ± standard errors of the percentage methylation are shown.

In another experiment, looking at additional time points after the second challenge (4, 12, 48, and 96 h after the second challenge), we still did not find DNA methylation in larvae exposed to the fungal pathogen. We found RNA methylation, but no significant differences were found among treatments (Priming vs. Control; *F* = 0.001, *p* = 0.97) or the interaction time^∗^treatment (*F* = 0.13, *p* = 0.94). The only difference in RNA methylation rate was dependent on time (*F* = 32.24, *p* < 0.0001; **Figure 6**) since it decreased significantly at 12 and 48 h after the second challenge compared to 4 and 96 h (**Figure 6**).

## Discussion

Our results confirmed the occurrence of immune priming both across generations (Moret, 2006; Zanchi et al., 2012) and within generations in adult *T. molitor* challenged with bacteria. In addition, we investigated the global methylation status for DNA and RNA for the first time in invertebrates during immune priming.

Methylation is found in various forms of RNA, including ribosomal, transfer, messenger, and several types of small RNAs, but the function of methylation remains unclear (Chow et al., 2007; Chen et al., 2016). We acknowledge that we extracted the total RNA and we do not know which types of RNAs were methylated. However, we speculate that the most likely form subject to methylation is ribosomal RNA, due to its abundance (Fromm et al., 2011; Yanez-Barrientos et al., 2013). The 5mC function in rRNA could be involved in translational fidelity, as quality control checkpoints in ribosome assembly and tRNA recognition (Song and Nazar, 2002; Chow et al., 2007; Motorin et al., 2010). In vertebrates, 5mC in tRNA is related to immune recognition (Kaiser et al., 2014), and although miRNA or small nuclear RNA (snRNA) may also be implicated, the function of such molecules is not well understood (Chen et al., 2016).

Independently of the specific mechanism and type of methylated RNA, we suggest a role for RNA methylation after the second challenge during immune priming within generations. This hypothesis may be plausible because (a) our results revealed that immune activation alone did not cause differential methylation (Experiment 2), (b) we observed a lower percentage of methylation in RNA during immune priming in the Priming group than in the Control group in both larvae and adults challenged with the fungus *M. anisopliae* and the bacterium *M. lysodeikticus* (Experiments 1 and 4), respectively, and (c) we only found differential methylation 24 h after the second immune challenge (Experiment 4), consistent with the peak of immune response after immune challenge in *T. molitor* (Haine et al., 2008; Johnston et al., 2014). However, we acknowledge that these results are still correlative and deserve further investigation. For example, our experiments were not fully factorial, i.e., a treatment group with an *M. lysodeiktikus*-PBS challenge is missing. In addition, we propose that the full kinetics of methylation in RNA before and after the first and second challenge, together with the knowledge of which form of RNA is methylated, will provide more information to address the importance on immune priming. This kinetics, as well as the gene expression during immune priming, deserves further investigation because the exact mechanism of immune priming remains unknown for all invertebrate species (Tate and Graham, 2015; Contreras-Garduño et al., 2016; Milutinović and Kurtz, 2016).

Epigenetics-based on changes on RNA requires non-Mendelian inheritance. Female and male parents could transmit information and a regulatory role for ncRNA methylation in gene expression and in RNA-dependent inheritance is suggested by this kind of inheritance (Liebers et al., 2014). Therefore, we predicted that a difference would exist in the percentage of 5mC between the Primed and Control animals across generations; however, this was not found in our data, and further analysis of other epigenetic markers are required to know if epigenetic mechanisms underlie immune priming across generations (Liebers et al., 2014). The same phenomenon may occur for 5mdC but we did not find evidence of it with our study in *T. molitor*. Our chromatographic procedure enables to assess a minimum of 0.06% of methylation for not less than 80 ng of DNA extracted (López-Torres et al., 2011), suggesting that if differential 5mdC did exist, it was below our detection threshold. Another explanation could be that DNA methylation may occur in some developmental stages but not others (Lyko et al., 2000; Field et al., 2004). For these reasons, we used both larvae and adults in our experiments. Another explanation may be that differences might be dose-dependent, but we did not find methylation in DNA by using single insects or pools of 5 or 10 animals (data not shown). Finally, it is possible that as no sequence-specific method was used, any subtle changes in certain genes will remain undetected in both immune challenge (injection of pathogens once) and immune priming (innate immune memory) across generations. Nevertheless, our results support another study with the tenebrionid *Tribolium castaneum*, which found extremely low levels of methylation of mitochondrial DNA and no detectable methylation of nuclear DNA (Zemach et al., 2010). Hence, if methylation in *T. molitor* is present at extremely low levels of total genomic DNA, it may occur exclusively in regulatory regions of some specific genes rather than throughout the entire genome (Field et al., 2004). Hence, the occurrence of 5mdC in different insect species remains to be tested during immune priming within and across generations, as well as the types of methyltransferases in *T. molitor*.

We acknowledge that other mechanisms could be implicated within and across generations, as for example, histone acetylation, micro RNAs (miRNAs) or RNA methylated at the six position of the adenosine base (m6A), and/or endoreplication. In both vertebrates and invertebrates, acetylation is important during immune responses and resistance (reviewed in Field et al., 2004; Mukherjee et al., 2012) and a recent and interesting study suggests that miRNAs could play a key role in immune priming across generations (Mukherjee and Vilcinskas, 2014). Endoreplication is another potential mechanism because it has been implicated in immune priming within generations (Contreras-Garduño et al., 2015). Although correlative, our work suggests that the mechanism involving specific or general RNA methylation merits further research to understand the mechanisms of immune priming. For example, we need to know which types of RNA are methylated, and why primed insects’ RNA is less methylated than that of controls deserves further research. In addition, which epigenetic mechanisms might be involved in the immune priming across generations other than DNA methylation, or why some but not other species show methylations of DNA and/or RNA merits more studies.

Finally, according to recent reports, different epigenetic mechanisms based on RNA may play a key role on organisms’ physiology (Liebers et al., 2014; Liu and Pan, 2015; Stunnenberg et al., 2015; Chen et al., 2016), and these mechanisms deserve further study to determine which mechanisms of immune priming act within and across generations, as well as during the animal’s immune response.

## Ethics Statement

Ethic committee of the Universidad of Guanajuato from Mexico. No humans were used.

## Author Contributions

CC-V and CL-L carried out the experiments with adults and larvae against bacteria and with larvae against fungus, respectively. KW, KeW, and AL-T analyzed methylations. JT-G, GH, HL-M and JC-G designed the experiments. All authors contributed to writing the manuscript.

## Conflict of Interest Statement

The authors declare that the research was conducted in the absence of any commercial or financial relationships that could be construed as a potential conflict of interest.
